# Substitution Mapping of a Locus Responsible for Hybrid Breakdown in Populations Derived From Interspecific Introgression Line

**DOI:** 10.3389/fpls.2021.633247

**Published:** 2021-04-21

**Authors:** Nilsa Emilia Munguambe, Shouta Inoue, Zita Demeter, Yoshiyuki Yamagata, Hideshi Yasui, Shao-Hui Zheng, Daisuke Fujita

**Affiliations:** ^1^Tropical Crop Improvement Laboratory, Faculty of Agriculture, Saga University, Saga, Japan; ^2^Plant Breeding Laboratory, Faculty of Agriculture, Kyushu University, Fukuoka, Japan

**Keywords:** rice, *Oryza meridionalis*, tiller number, substitution mapping, hybrid breakdown

## Abstract

Hybrid breakdown, a form of postzygotic reproductive barrier, has been reported to hinder gene flow in many crosses between wild and cultivated rice. Here, the phenomenon of hybrid breakdown was observed as low-tillering (i.e., low tiller number) in some progeny of an interspecific cross produced in an attempt to introduce *Oryza meridionalis* Ng (W1625) chromosomal segments into *Oryza sativa* L. ssp. *japonica* “Taichung 65” (T65). Low-tillering lines were obtained in BC_4_-derived progeny from a cross between W1625 and “Taichung 65,” but the locus for low-tillering could not be mapped in segregating populations. As a second approach to map the locus for low-tillering, we analyzed an F_2_ population derived from a cross between the low-tillering lines and a high-yielding *indica* cultivar, “Takanari.” A major QTL for low-tillering, *qLTN4*, was detected between PCR-based markers MS10 and RM307 on the long arm of chromosome 4, with a LOD score of 15.6. The low-tillering phenotype was associated with weak growth and pale yellow phenotype; however, low-tillering plant had less reduction of grain fertility. In an F_4_ population (4896 plants), 563 recombinant plants were identified and the low-tillering locus was delimited to a 4.6-Mbp region between markers W1 and C5-indel3729. This region could not be further delimited because recombination is restricted in this region of *qLTN4*, which is near the centromere. Understanding the genetic basis of hybrid breakdown, including the low-tillering habit, will be important for improving varieties in rice breeding.

## Introduction

Panicle number in rice (*Oryza sativa* L.) is a yield component that is directly influenced by tillering ability during the growth period. Tillers are additional culms (stems) that develop from the main culm and are similar to branches (Smith and Dilday, 2003). Tiller number is an important trait for improving rice varieties in breeding programs. In the late 1980s, new plant type (NPT) breeding was launched at the International Rice Research Institute (IRRI, Los Banos, Philippines) to increase rice yield potential. One of the target traits for the NPT was the low-tillering trait (i.e., low number of tillers) because low-tillering varieties have fewer unproductive tillers (Janoria, 1989). Rice varieties with high tiller number are generally suitable under some conditions; however, excessive tillering can lead to yield reductions due to an increased number of unproductive tillers. Under wet direct-seeding conditions or in locations where drought is expected to occur episodically, desirable genotypes are expected to have few but vigorous tillers (Fujita et al., 2010). Therefore, the development of rice varieties with optimal tiller number for a specific environment could play an important role in increasing production (Kebrom et al., 2012).

Numerous studies have reported that tiller and panicle number (PN) is controlled by multiple genes (Zhu et al., 2011; Hussien et al., 2014). Across these studies, several major genes for tiller number, initially identified through mutant phenotypes, have been isolated and validated. The gene *TEOSINTE BRANCHED-1*, which negatively regulated lateral branching, was detected on chromosome 3 (Takeda et al., 2003). *MONOCULM 1*, which encodes a putative GRAS family nuclear protein, was located on chromosome 6 and promotes the formation of axillary buds (Li et al., 2003). Two genes for reduced culm number, *RCN8* and *RCN9*, have been mapped on the long arm of chromosome 1 and the long arm of chromosome 6, respectively (Jiang et al., 2006). The *Ltn* (low tiller number) gene from “Aikawa 1” was detected on the long arm of chromosome 8 (Fujita et al., 2010), and *ltn2* from NPT rice was identified on chromosome 7 at a distance of 2.1 cM from the SSR marker RM21950 (Uddin et al., 2016).

The use of exotic germplasm, including wild rice species, is expected to extend the available genetic variation in cultivated rice. To facilitate improvement of disease and insect resistance, resistance genes from wild rice species have been introduced into cultivated rice varieties (Khush, 1997). However, the identification of genes from wild rice species is difficult because of reproductive barriers between rice cultivars and wild rice species. Thus, there are few reports on exploitation of genes from wild rice species related to agronomic traits such as tiller number. To identify potentially useful genetic factors in wild rice species, Yoshimura et al. (2010) developed a set of introgression lines (ILs) carrying wild donor chromosomes segments in an uniform genetic background. The ILs are important materials for precisely evaluating the phenotype of each plant and fine-mapping genes as single Mendelian factors.

As mentioned above, the introgression of agronomically useful genes from wild rice into cultivated rice is often prevented by reproductive barriers. Hybrid breakdown, a type of postzygotic reproductive barrier, is defined as weakness or sterility in the F_2_ or later generations (Fukuoka et al., 1998; Yamamoto et al., 2007, 2010; Miura et al., 2008; Kubo, 2013). In previous studies, genes inducing hybrid breakdown have been reported in both inter- and intraspecific crosses of AA genome species. The AA genome wild rice is considered to be the direct ancestor of cultivated rice. The *hybrid weakness f-1* gene from *O*ryza *glumaepatula* was located on the short arm of chromosome 4 and found to induce hybrid breakdown in the genetic background of *japonica* rice variety “Taichung 65” (T65) (Sobrizal and Yoshimura, 2009). The *hbd1(t)* gene from *O*ryza *nivara* was located on the short arm of chromosome 2 and produced a similar hybrid breakdown phenotype in the “Koshihikari” genetic background (Miura et al., 2008). To our knowledge there are no other reports of hybrid breakdown genes involving other AA-genome species.

To broaden the available rice genetic resources from wild species, *O. meridionalis* (AA genome), a wild rice that is endemic to Oceania, New Guinea, and Australia (Sotowa et al., 2013) was used to develop 36 ILs with introduced chromosome segments of *O. meridionalis* in the genetic background of T65 (Yoshimura et al., 2010). Among these ILs, we identified one line with yellow leaves and greatly reduced tiller number. Through analysis of a segregating population derived from a cross between the low-tillering IL and T65, we identified a locus related to low-tillering but could not map it in that population. By crossing the low-tillering line to another cultivar (“Takanari”), producing segregating populations, and performing substitution mapping, we delimited a major QTL for low-tillering.

## Materials and Methods

### Plant Materials Containing Low-Tillering Trait in T65 Genetic Background

**FIGURE 1:**
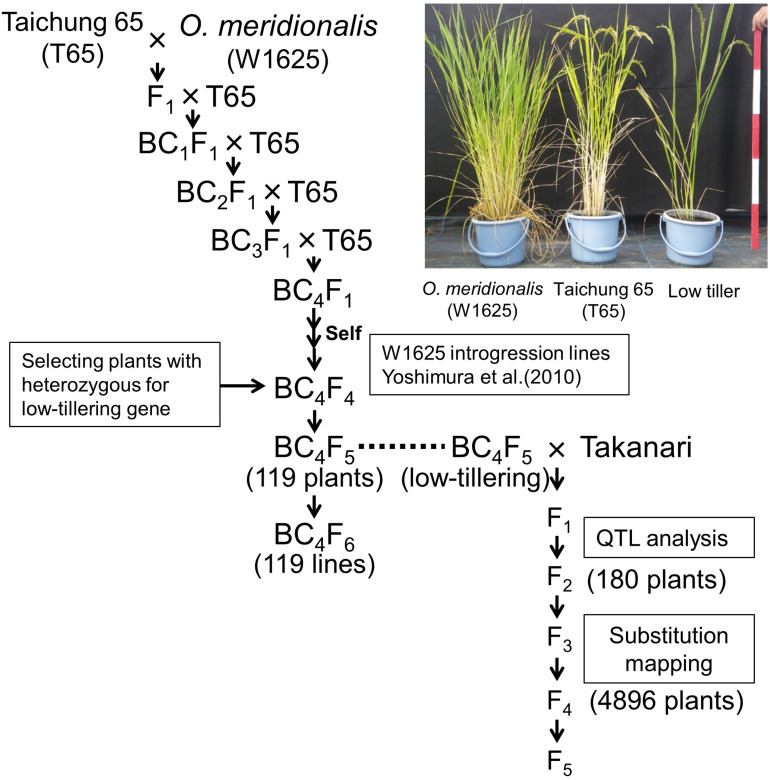
Breeding scheme for developing lines with low-tillering. The BC_4_F_5_ and BC_4_F_6_ populations were derived from a cross between “Taichung 65” (T65) and *O. meridionalis*. The F_2_ population was derived from a cross between low-tillering BC_4_F_5_ plants and “Takanari.”

In a previous study *O. meridionalis* (W1625) was crossed with *O. sativa* ssp. *japonica* “Taichung 65”, and the F_1_ plants were repeatedly backcrossed to T65 as the recurrent parent (Figure 1; Yoshimura et al., 2010). A set of W1625 ILs (36 lines) in BC_4_F_4_ was selected by marker-assisted selection (MAS) (Yoshimura et al., 2010). One line among the W1625 ILs showed segregation of a particular phenotype with weaker growth (low-tillering and yellow leaves) compared with normal plants. To identify the locus controlling low tiller number, a BC_4_F_5_ segregating population (derived from a single self-pollinated BC_4_F_4_ plant) was used for genetic analysis. To confirm the genotype at the low-tillering locus for each BC_4_F_5_ plant, the corresponding BC_4_F_6_ lines were used for progeny tests. A set of 119 BC_4_F_5_-derived lines was used for analyzing the introgressed chromosomal segments with SSR markers. BC_5_F_1_ plants derived from a cross between T65 and low-tillering plant (BC_4_F_5_) were used for confirming the effect of the low-tillering trait on other agronomic traits. The BC_5_F_2_ plants were evaluated for tillering number and panicle structure.

### Plant Materials for Mapping the QTL

To map the locus for low-tillering, we developed an F_2_ population derived from a cross between the low-tillering plant (BC_4_F_5_) and a high-yielding *indica* cultivar, “Takanari.” The F_2_ plants were self-pollinated to generate F_3_, F_4_, and F_5_ lines for substitution mapping of the low-tillering locus. F_2__:__3_ and F_4__:__5_ lines were used for progeny tests to confirm the genotypes at the low-tillering locus of the F_2_ and F_4_ plants, respectively.

### Characterization of Agronomic Traits in Normal- and Low-Tillering Lines

All seeds were incubated at 25°C for 3 days to induce germination and then sown in seeding trays. The seedlings were transplanted to the paddy field at Saga University (Saga, Japan) at 30 days after sowing and planted one plant per hill. The BC_4_F_5_ population was planted with 18 cm between plants within a row and 30 cm between rows in an experimental field at Kyushu University (Kasuya, Fukuoka, Japan). The other materials (BC_4_F_6_, BC_5_F_1_, BC_5_F_2_, F_2_, F_3_, F_4_, and F_5_) were planted with 20 cm between plants within a row and 25 cm between rows in an experimental field at Saga University (Saga, Japan). The PNs per plant were observed at the maturity stage and measured in all segregating populations. In the F_3_, and F_5_ generations, which were used for progeny tests (using 20 plants of each entry), plants with fewer than 5 panicles were considered as low-tillering. To examine tiller number after transplanting, 10 plants per entry were transplanted to 5-L pots and tiller numbers were counted every week. To understand the effects on other agronomic traits caused by low-tillering locus, the BC_4_F_6_ and BC_5_F_1_ plants (10 plants of each entry) were evaluated for culm length (CL), panicle length (PL), PN, leaf length (LL), leaf width (LW), number of primary branches (NPB), number of secondary branches (NSB), total spikelet number per panicle (TSN), grain fertility per panicle (GF), total number of spikelets on the primary branch (TSPB), and total number of spikelet on the secondary branch (TSSB).

### DNA Extraction and Genotyping

The genomic DNA of parents and segregating populations was extracted from freeze-dried leaves by using the potassium acetate protocol (Dellaporta et al., 1983). In preparation for extraction, the leaves were collected in 96-well deep-well plates and crushed using a FastPrep 96 (MP Biomedicals, United States). DNA marker genotypes were determined by PCR with a Gene Atlas thermal cycler (ASTEC, Japan). PCR amplification conditions consisted of 96°C for 5 min; followed by 35 cycles of 96°C for 5 min, 55°C for 30 s, and 72°C for 30 s; followed by a final extension of 25°C for 1 min. The PCR products were separated by electrophoresis in 4% agarose gel, stained with ethidium bromide, and viewed under ultraviolet light.

### Construction of the Linkage Map and QTL Analysis

The DNA markers were first screened for polymorphism between the low-tillering line and “Takanari” and then used for genotyping the F_2_ populations. The genetic map for the F_2_ population was constructed using 113 markers that were polymorphic between the parents and distributed across all 12 chromosomes. In this map, genetic distances were determined using the Kosambi function. The QTL analysis was performed by Windows QTL Cartographer software version 2.5 and conducted using data for PN and DNA marker genotypes (Wang et al., 2012). On the basis of a 1000-permutation test, a LOD score greater than 3.6 was considered as the threshold at a 0.05 level of significance.

### Substitution Mapping of the Low-Tillering Locus

To determine the precise location of the low-tillering locus, the tillering phenotypes and genotypes of DNA markers around the low-tillering locus in 180 F_3_ lines were used to confirm the genotypes at that locus in the F_2_ population. To further delimit the location of the low-tillering locus, a large segregating F_4_ population (4896 plants) derived from F_3_ lines heterozygous at the low-tillering locus were screened with markers RM8213 and RM307 to select recombinant plants. The genotypes of recombinant plants were analyzed using nine SSR markers (RM16459, RM16502, RM16535, RM16550, RM16605, RM16626, and RM307) and three Indel markers (RH7, W1, C5-indel3729, C5-indel3743, and C5-indel3757) around the low-tillering locus (Supplementary Table 1). Finally, an F_5_ progeny test was performed to confirm the genotype of the low-tillering locus for each F_4_ individual.

## Results

### Identification of the Low-Tillering Locus

**FIGURE 2:**
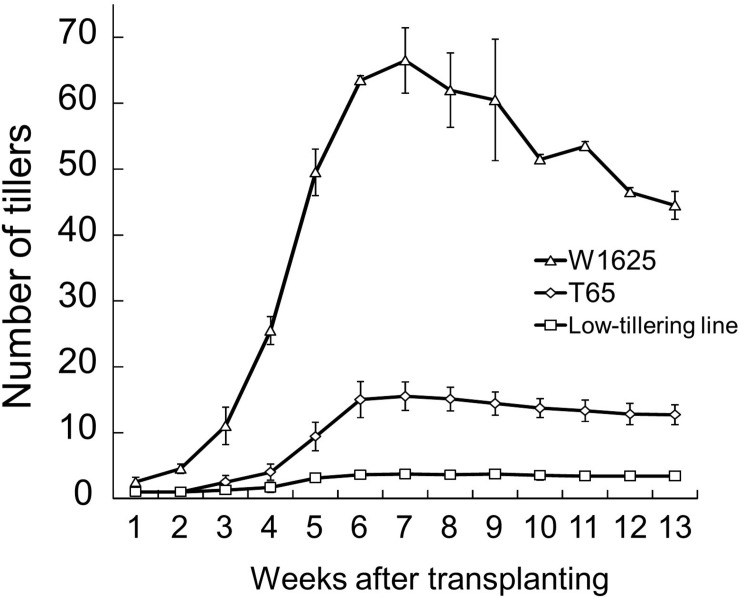
Fluctuation in tiller number during plant development in W1625, T65, and the low-tillering line. Data are means ± SD (*n* = 10).

The tiller numbers per plant of W1625, T65, and a low-tillering line (BC_4_F_6_ generation which seems to have had homozygous low-tillering lines) were evaluated every week during the tillering stage (Figure 2). Throughout the tiller development stage, the low-tillering line continually showed few tillers. The PNs at maturity of W1625, T65, and the low-tillering line were 44, 13, and 4, respectively. The number of panicles per plant of W1625 was higher than that of T65 at the maturing and greatly reduced in the low-tillering line compared with both parents. These data suggested that the total number of panicles in the low-tillering line (with T65 genetic background) was affected by one or more introgressed segments from W1625.

**FIGURE 3:**
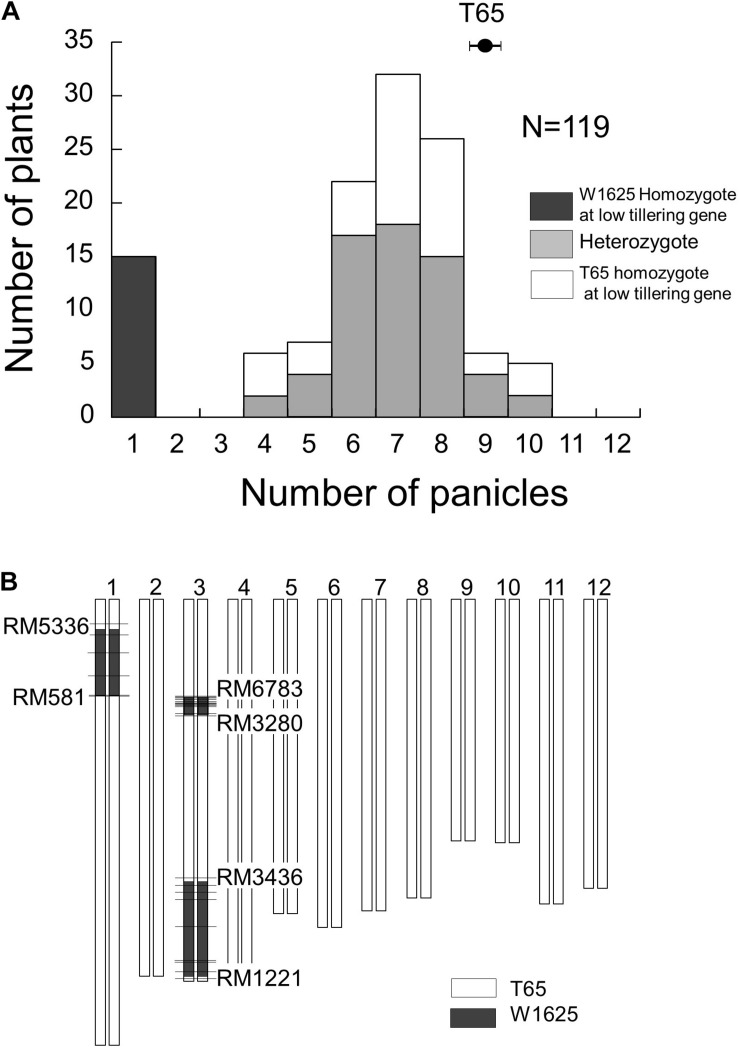
Frequency distribution of panicle number **(A)** and graphical genotype of low-tillering line **(B)** in BC_4_F_5_ population derived from a cross between T65 and W1625. Black, gray, and white colors in **(A)** indicate W1625 homozygote, heterozygote, and T65 homozygote for low-tillering locus through scored based on segregation of phenotypes in the next generation. The horizontal bars in **(B)** show the location of polymorphic SSR markers.

To identify the locus for low-tillering, the BC_4_F_5_ segregating population was analyzed. The frequency distribution of PN in that population showed a bimodal distribution: 15 plants with low-tillering (one panicle per plant) and 104 plants with PN ranging from 4 to 10 (Figure 3A). To elucidate the genotypes at the low-tillering locus in the BC_4_F_5_ segregating population, the PNs in BC_4_F_6_ lines were observed as a progeny test. Among the 119 BC_4_F_5_ plants, 15 plants were homozygous for the W1625 allele at the low-tillering locus (progeny were all low-tillering), 60 plants were heterozygous (progeny segregated for low-tillering phenotype), and 44 plants were homozygous for the T65 allele (progeny were all normal tillering). The segregation ratio in the BC_4_F_5_ population (15:60:44) did not fit the 1:2:1 expected ratio (χ^2^ = 14.1, *P* = 0.001).

### Identification of W1625 Chromosomal Segments Retained in the BC_4_F_5_ Population

The chromosomal constitution of the low-tillering line was assessed by using 492 SSR markers (Figure 3B). There were three introgressed segments from W1625 detected in the low-tillering line: one on the short arm of chromosome 1 and two on chromosome 3. Additionally, through genotyping by sequencing, three large introgressed segments from W1625 on the low-tillering line were identified at same regions using 5445 SNPs and there are three small regions introgressed segment from W1625 on chromosomes 4, 10, and 12 (Supplementary Figure 1). There was no association between tiller number and genotype of these three large W1625 segments in the BC_4_F_5_ segregating population. In BC_5_F_2_ segregating population, there are no association between agronomic traits (PN, TSN, and SNB) and genotype of these three large W1625 segments and the TSN and SNB of BC_5_F_2_ plants with low-tillering showed lower than that of T65 (Supplementary Figure 2). These results suggested that other chromosomes might harbor small introgressed segments from W1625 that are related to the low-tillering phenotype, TSN and SNB.

### Detection of a QTL for Panicle Number

**FIGURE 4:**
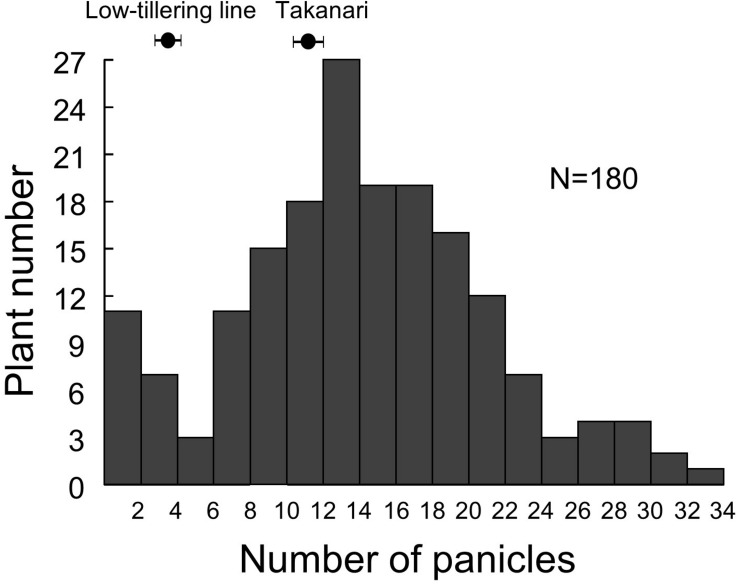
Frequency distribution of panicle number in F_2_ population derived from a cross between low-tillering plants and “Takanari.”

Because we were unable to detect the location of the low-tillering locus in the BC_4_F_5_ population, we developed an F_2_ population from a cross between the low-tillering line and “Takanari” to conduct QTL analysis. The PNs of the parents were 4 for the low-tillering line and 11 for “Takanari” (Figure 4). The frequency distribution for PN per plant in the F_2_ population showed a continuous distribution with approximately 60% of the individuals having a higher PN than the parents. To estimate the location of the QTL for PN, a QTL analysis was conducted using this F_2_ population. A QTL designated *qLTN4* (QTL for low tiller number on chromosome 4) was detected between MS10 and RM307 on the short arm of chromosome 4 (Table 1). This QTL had a LOD score of 15.6 and explained 30.4% of the phenotypic variation for PN. The allele from the low-tillering line at *qLTN4* was associated with decreased PN per plant. Additionally, five regions on chromosomes 5, 6, 8, 10, and 11 had LOD peaks and LOD score is less than threshold of LOD score at 5% level. The alleles from the low-tillering line at two QTLs, *qLTN5*, and *qLTN6*, showed increasing PN per plant, while the alleles from the low-tillering line at three QTLs, *qLTN8, qLTN10*, and *qLTN11*, showed decreasing PN per plant.

**TABLE 1:** The QTL for tiller number identified in an F_2_ population derived from a cross between “Takanari” and low-tillering line.

QTL	Chromosome	Marker interval	Peak LOD score	PVE (%)^a^	Additive effect^b^	Dominance effect^b^
*qLTN4*	4	MS10-RM307	15.6	30.4	7.4	5.5
*qLTN5*	5	RM249-RM430	2.9	5.4	−1.9	−1.8
*qLTN6*	6	RM6395-RM1370	1.6	2.8	−1.8	0.4
*qLTN8*	8	RM1345-RM25	2.4	6.2	2.5	−0.5
*qLTN10*	10	RM1873-RM496	1.8	3.2	1.3	−1.6
*qLTN11*	11	RM6091-RM229	2.5	4.4	0.7	2.8

*The QTLs for panicle number has more than 1.5 of LOD score. ^*a*^Proportion of the phenotypic variation explained by QTL. ^*b*^Values indicate the relative effect of the “Takanari” allele.*

### Substitution Mapping of *qLTN4*

**FIGURE 5:**
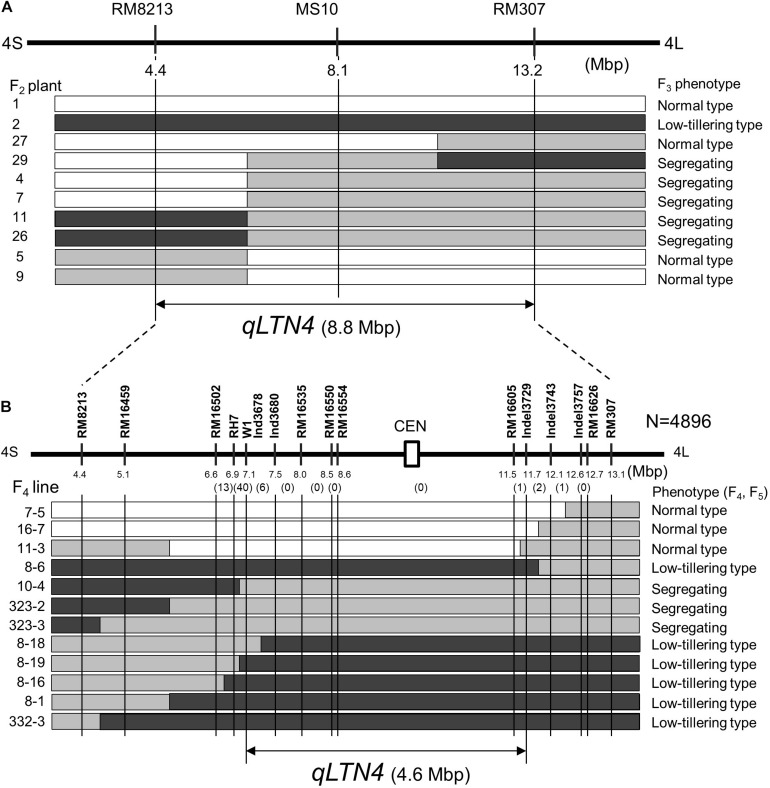
Substitution map of locus for low tiller number. **(A)** Substitution mapping of *qLTN4* in F_2_ plants and F_3_ lines derived from a cross between “Takanari” and the low-tillering line. **(B)** Substitution mapping of *qLTN4* in F_4_ and F_5_ lines. Black, gray, and white indicate regions homozygous for marker allele from low-tillering line, heterozygous, and homozygous for marker allele from “Takanari.” The white rectangle with CEN indicate centromere region of chromosome 4.

To delimit the location of *qLTN4*, a progeny test was performed using F_3_ lines. These lines, derived from F_2_ plants with recombination between markers RM8213 and RM307, were classified into three phenotypes: low-tillering fixed, segregating, and normal-tillering fixed. By comparing F_2_ genotypes and F_3_ phenotypes, *qLTN4* was located within an 8.8-Mbp interval between markers RM8213 and RM307 (Figure 5A). To further refine the location of *qLTN4*, recombinant plants were selected from 13 F_4_ families with heterozygosity at *qLTN4*. Among a large segregating population (4896 F_4_ plants), 563 plants with a recombination event between markers RM8213 and RM307 were selected. These 563 plants were evaluated for PN and genotyped using PCR-based markers. Based on the phenotypes and genotypes at each marker for 13 recombinant plants, lines ISC11-3 and ISC8-18, with recombination that took place between markers W1 and C5-indel3729, delimited the low-tillering locus to a 4.6-Mbp region (Figure 5B).

### Characterization of Agronomic Traits Affected by *qLTN4* on Low-Tillering Line

To determine the influence of *qLTN4* on agronomic traits, we characterized panicle architecture and other traits in the low-tillering line (BC_4_F_6_). CL and PN, of the low-tillering line were significantly lower than those of T65 despite their similar genetic background (Table 2). On the other hand, LW was significantly larger in the low-tillering line than in T65 or the BC_5_F_1_. BC_5_F_1_ plants derived from a cross between T65 and the low-tillering line had similar agronomic traits to T65 with the exception of PL, which was significantly larger in the BC_5_F_1_. There were no significant differences for CL, PN, LL, and LW between T65 and BC_5_F_1_ plants. Several traits related to panicle architecture, i.e., TSPB, TSSB, and TSN, were significantly lower in the low-tillering line than in T65. The grain fertility of the low-tillering line was 75.4%, significantly lower than that of T65 (94.5%; Table 3). However, NPB, NSB, and TSPB of the BC_5_F_1_ were not significantly different from those of the low-tillering line.

**TABLE 2:** Agronomic traits of “Taichung 65” (T65), BC_5_F_1_ (T65/low-tillering line), and low-tillering line.

Line	CL (cm)	PL (cm)	PN	LL (cm)	LW (cm)
Taichung 65 (T65)	99.6 ± 3.6a	19.8 ± 1.8a	13.0 ± 3.6a	42.8 ± 4.2a	1.41 ± 0.09a
BC_5_F_1_ (T65/Low-tillering line)	102.2 ± 4.3a	24.4 ± 0.6b	9.8 ± 1.9a	39.0 ± 3.5a	1.44 ± 0.09a
Low-tillering line	67.6 ± 4.4b	20.5 ± 1.3a	3.8 ± 1.7b	37.9 ± 5.3a	1.70 ± 0.09b

*Data are means ± SD (*n* = 10). The different alphabetical letters indicate significant difference (*p* < 0.05, Tukey Kramer test).*

**TABLE 3:** Panicle structure of “Taichung 65” (T65), BC_5_F_1_ (T65/low-tillering line), and low-tillering line.

Line	NPB	NSB	TSPB	TSSB	TSN	GF (%)
Taichung 65 (T65)	12.0 ± 1.1a	34.5 ± 6.3a	68.7 ± 7.1a	102.9 ± 23.5a	171.5 ± 24.2a	94.5 ± 3.5
BC_5_F_1_ (T65/Low-tillering line)	10.7 ± 2.9a	34.1 ± 10.1a	58.1 ± 15.6b	109.3 ± 33.9a	167.3 ± 48.1a	ND^1^
Low-tillering line	10.1 ± 1.6a	22.0 ± 5.4a	55.6 ± 7.0b	63.4 ± 18.2b	118.9 ± 23.2b	75.4 ± 10.3

*Data are means ± SD (*n* = 5). ^1^The BC_5_F_1_ plants were not observed grain fertility. The different alphabetical letters indicate significant difference (*p* < 0.05, Tukey Kramer test).*

## Discussion

Tillering, i.e., the production of multiple stems, is an important agronomic trait that has been widely studied in cereals. In previous studies, genes and QTLs with large effects on tiller number were mapped in various regions of the rice genome. *MONOCULM 1* was located on chromosome 6 (Li et al., 2003), *Ltn* from “Aiwaka 1” on chromosome 8 (Fujita et al., 2010), and *ltn2* from an NPT variety on chromosome 7 (Uddin et al., 2016). Among the QTLs and genes for tiller number on chromosome 4, *tn4-1* was located between markers *RG190* and *RG908* and was detected by the conditional QTL method (Yan et al., 1998). The QTL detected in the present study, *qLTN4*, was detected on chromosome 4 and had a large contribution to the phenotypic variation (30.4%) and high LOD score (15.6). The chromosomal region of *qLTN4* overlapped with that of *tn4-1* (Yan et al., 1998), suggesting that *qLTN4* might be same QTL.

In a previous study, segregation distortion as a consequence of nuclear and cytoplasmic factors and zygotic selection was observed in two reciprocal F_2_ crosses and a BC_1_ population (Reflinur et al., 2014). Another report mentioned that segregation distortion was caused by other factors such as population size, genotyping errors, and some non-biological factors (Falconer and Mackay, 1996). Furthermore, among different cross combination, different factors may contribute to segregation distortion (Xu et al., 1997). In the present study, the distribution of PN in the BC_4_F_5_ segregating population showed a slightly bimodal distribution but did not fit the expected 3:1 ratio. In the F_2_ population, significant segregation distortion was observed at MS10 (near *qLTN4*), reducing the frequency of low-tillering plants. Similarly, the low-tillering gene locus segregated in the BC_4_F_5_ population as 44 homozygous for the T65 allele, 60 heterozygous and 15 homozygous for the W1625 allele. This same tendency for segregation distortion around *qLTN4* in both F_2_ and BC_4_F_5_ populations is evidence for segregation distortion in this region.

The low-tillering lines were characterized in different growth conditions. In BC_4_F_5_ population, the plants with 18 cm between plants within a row and 30 cm were grown in Fukuoka, JAPAN. In BC_4_F_6_, BC_5_F_1_, F_2_, F_3_, F_4_, and F_5_, the plants with 20 cm between plants within a row and 25 cm were grown in Saga, JAPAN. The PN of low-tillering plant is one in BC_4_F_5_ population (Figure 3A), while the PN of low-tillering line of BC_4_F_6_ is 3.8 (Table 2). The difference suggested that the PN of low-tillering line is influenced by environmental factors such as transplanting interval. Therefore, we defined plants with fewer than 5 panicles as low-tillering in progeny test (F_3_ and F_5_ those were grown in Saga, JAPAN)

The low-tillering line was analyzed for detecting introgression segments from W1625. Based on SSR markers, three segments were identified on chromosomes 1 and 3. However, the chromosome segment from W1625 around the *qLTN4* region on the low-tillering line was not detected. Therefore, through genotyping by sequencing, 5445 SNPs was used for detecting W1625 chromosomal segment on low-tillering line. Three large segments on chromosomes 1 and 3 and three additional small segment on chromosomes 4, 10, and 12 have been detected. However, the location of small chromosomal segment of W1625 on chromosome 4 is outside *qLTN4* region. Furthermore, we read whole genome sequence of low-tillering lines and several SNPs from W1625 detected around *qLTN4* region (Supplementary Figure 3). However, we cannot find exact evidence of association between low-tillering and W1625 chromosomal segment in this area. Therefore, these results suggested that there are possibilities for small introgression segment of W1625 locating on this region or natural mutation of low tiller line. The SNPs and NGS analysis were conducted based on Nipponbare genome sequence, therefore, we might not detect W1625 chromosomal segments that are absent in Nipponbare genome. In future study, *de novo* sequence of W1625 would be necessary for detecting W1625 chromosomal segments. Also, the detection of *qLTN4* on earlier generation of segregation population derived from cross between T65 and W1625 would be possible to confirm association between low-tillering and W1625 chromosomal segments.

Meiotic recombination, which is the exchange of DNA between homologous chromosomes, is a fundamental process in eukaryotic reproduction (Dreissig et al., 2019). Previous studies have reported that recombination rates vary along chromosomes in most organisms with large genomes. High recombination rates were found near the distal ends of the chromosomes while low recombination rates were found in the region surrounding the centromere (Stapley et al., 2017; Haenel et al., 2018). Here, *qLTN4* was detected near the centromeric region of chromosome 4. In substitution mapping of *qLTN4*, we failed to narrow down the candidate region due to difficulty in finding recombinant plants in the F_3__:__4_ generation. This difficulty might have been caused by recombination restriction around the centromeric region of chromosome 4. In 4896 plants those were segregated at *qLTN4*, there is region for recombination restriction between RM16535 (8.0-Mbp) and RM16605 (11.5-Mbp) and we cannot obtain any recombinant plants between RM16535 and RM16605. Additionally, the genotypes of *qLTN4* through progeny test were completely linked to those of RM16535 and RM16605. To further delimit the *qLTN4* genomic region and identify candidate genes, expanding segregating population will be essential in a future study.

Hybrid breakdown has been described as sterility and weakness in F_2_ or later generations resulting from inter- or intraspecific crosses and can affect all aspects of plant development. Several studies have reported plants expressing various phenotypes such as sterility; severe reduction in plant height, tiller number, root number, and root length; or even plant death before heading time (Sato and Morishima, 1988; Kubo and Yoshimura, 2002; Miura et al., 2008). Yamamoto et al. (2010) observed two types of weak plants, severe and mild, in the F_2_ progeny of a cross between “Koshihikari” and “Habataki.” In the present study, the low-tillering plants (BC_4_F_5_) derived from a cross between T65 and *O. meridionalis* showed severe reduction in PN but only moderate reduction in other panicle traits, CL, and grain fertility compared with the recurrent parent. The phenotypes of the low-tillering line were considered to be caused by hybrid breakdown because they were observed in later generations derived from crosses between normal parental lines. We consider the type of hybrid breakdown that occurred in this study as the mild type described by Yamamoto et al. (2010). The low-tillering locus on the short arm of chromosome 4 was associated with reduction in CL, PL, PN, LW, NSB, and TSN. In a previous study by Sobrizal and Yoshimura (2009), the *hybrid weakness f-1* gene, associated with hybrid breakdown in a cross between T65 and *O. glumaepatula*, was located between G3006 and C933 (12.7-Mbp) on the short arm chromosome 4 and was completely linked to C708 (6.3-Mbp), C802 (6.9-Mbp), and R288 (8.2-Mbp). The *hybrid weakness f-1* gene showed a similar phenotype to *qLTN4* in the genetic background of T65. The *qLTN4* was located between W1 (7.1-Mbp) to Indel3729 (11.7-Mbp) and was overlapped with the regions of *hybrid weakness f-1* gene. Thus, we infer that the locus identified in our study might be the same locus that was previously identified by Sobrizal and Yoshimura (2009). Several studies have reported distant relationships between *O. meridionalis* and other AA-genome rice species (Second, 1986; Noredo et al., 1998). These results suggest that various AA-genome rice species may share a conserved region around *qLTN4* that is related to hybrid breakdown.

Several studies have proposed that interactions between complementary genes derived from each parent are the cause of hybrid weakness and hybrid breakdown (Fukuoka et al., 1998; Kubo and Yoshimura, 2002; Ichitani et al., 2007, 2011). In our study, only one locus involved in hybrid breakdown was detected. Assuming that hybrid breakdown in this case was also caused by the interaction of two complementary genes, the hybrid breakdown gene from T65 remains to be identified. Miura et al. (2008) were able to identify the gene responsible for hybrid breakdown in *O. nivara*; however, the complementary gene for hybrid breakdown from “Koshihikari” was not identified, suggesting a possible complex mechanism controlling this trait.

Hybrid breakdown has been reported as a hindrance to gene flow in many plant crosses. In this study, we observed a beneficial trait, low-tillering frequency, as a result of hybrid breakdown. Because the effects on other agronomic traits were mild, the allele with low-tillering on chromosome 4 might be useful for rice genetic improvement via production of low-tillering lines in breeding programs.

## Data Availability Statement

The original contributions presented in the study are included in the article/Supplementary Material, further inquiries can be directed to the corresponding author/s.

## Author Contributions

NM, SI, and DF designed the research. DF, NM, SI, and ZD performed the research. S-HZ, YY, and HY provided advice on the experiments. NM and DF wrote the manuscript. All authors contributed to the article and approved the submitted version.

## Conflict of Interest

The authors declare that the research was conducted in the absence of any commercial or financial relationships that could be construed as a potential conflict of interest.
