# Supplementary material for: Substitution Mapping of a Locus Responsible for Hybrid Breakdown in Populations Derived From Interspecific Introgression Line
Source: Front Plant Sci. 2021 Apr 21;12:633247. doi: 10.3389/fpls.2021.633247 (PMC8097182; doi:10.3389/fpls.2021.633247)
Supplement: Supplementary file 1 [file Presentation_1.pdf]

Supplementary Figure legends

Supplementary Figure S1. The graphical genotype of low-tillering line. The graphical genotype was constructed by using 5445 SNPs through genotyping by sequencing. The gray and white color indicate W1625 and T65 homozygote, respectively. The red color of bar indicated *qLTN4* region (4.6-Mbp). The blue line indicate the SNPs region from W1625 by whole genome sequencing.

Supplementary Figure S2. Panicle traits on BC_5F_2 plants derived from cross between BC_4F_5 plants and T65. A: Spikelet number per panicle and B: secondary branch number on BC_5F_2 plants those were categorized by phenotype and genotypes of SSR markers on chromosomes 1 and 3.

Supplementary Figure S3. The frequency distribution of number of SNPs from W1625 in low-tillering line. This physical distance is overlapped to *qLTN4* region (4.6-Mbp) on chromosome 4.

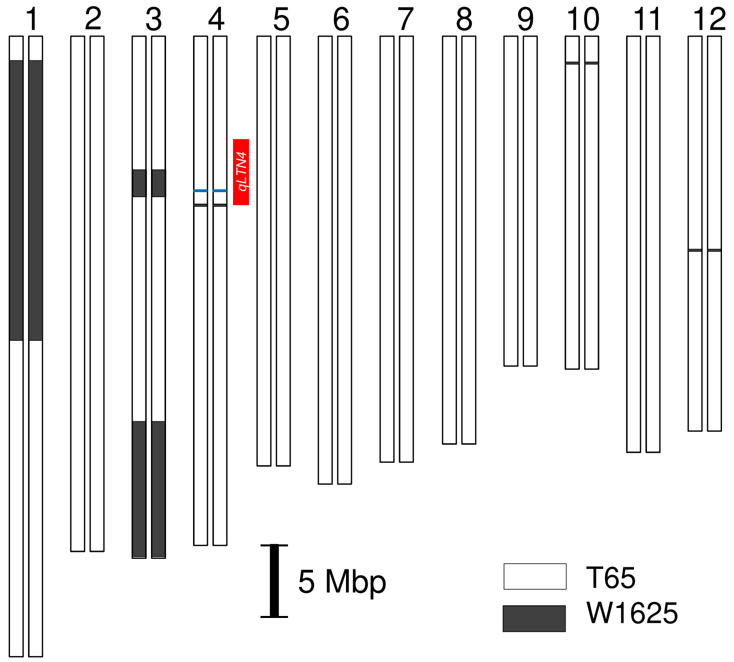

Supplementary Figure S1.

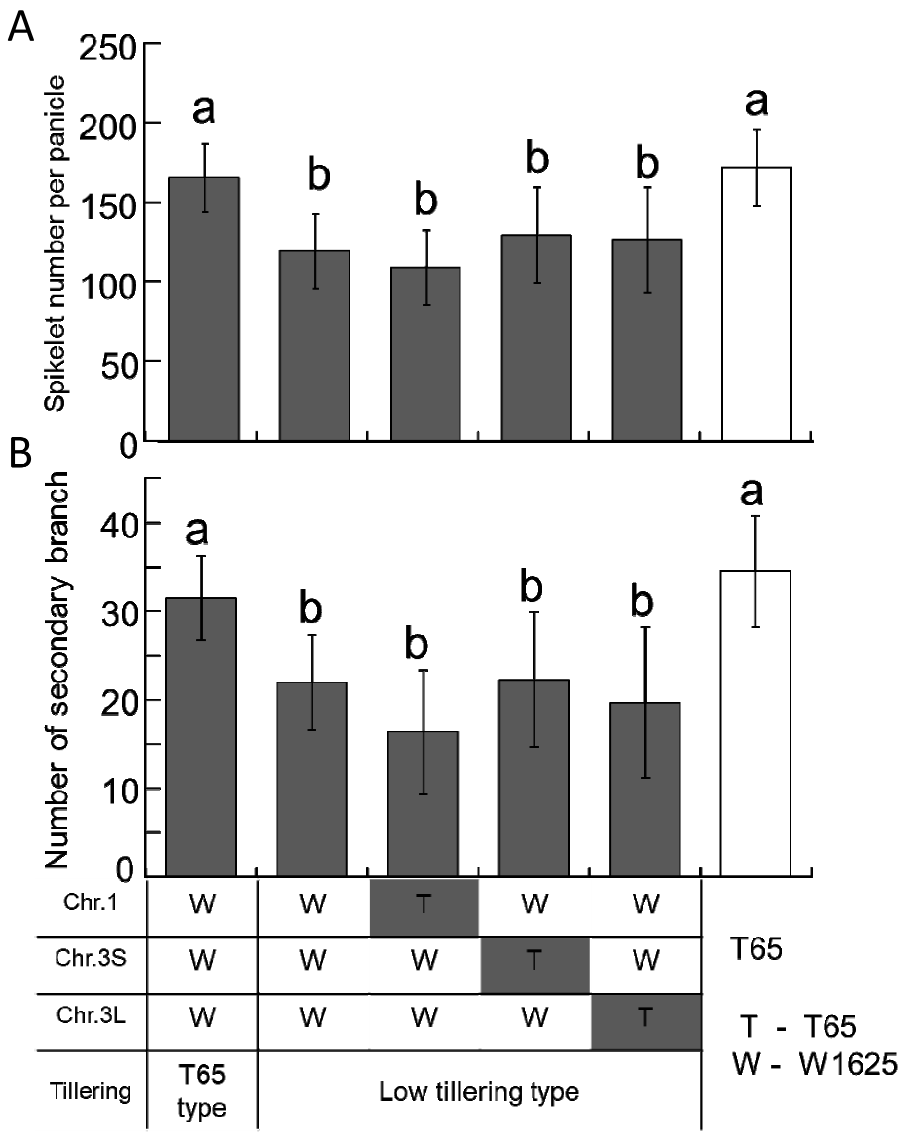

Supplementary Figure S2.

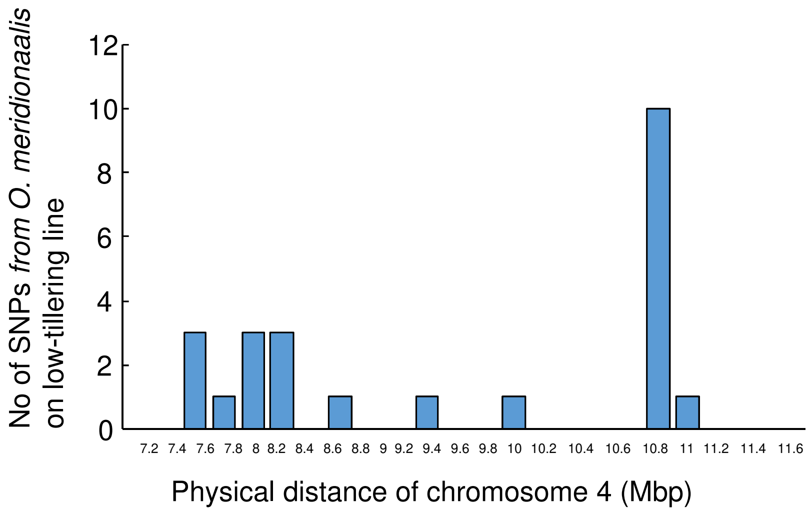

Supplementary Figure S3.
