# Supplementary material for: Substitution Mapping of a Locus Responsible for Hybrid Breakdown in Populations Derived From Interspecific Introgression Line
Source: Front Plant Sci. 2021 Apr 21;12:633247. doi: 10.3389/fpls.2021.633247 (PMC8097182; doi:10.3389/fpls.2021.633247)
Supplement: Supplementary file 2 [file Table_1.pdf]

Supplementary Table 1. DNA markers used for substitution mapping of low-tillering locus.

Marker	Forward primer sequence (5′ → 3′)	Reverse primer sequence (5′ → 3′)	Reference
RM8213	TGTTGGGTGGGTAAAGTAGATGC	CCCAGTGATACAAAGATGAGTTGG	McCouch et al. (2002)
RM16459	TCCAGGAGTTTGCCTTGTAGTGC	TAGCGAAGTCAGGATGGCATAGG	IRGSP (2005)
RM16502	GATGCCACATATCAGCTTGAGAGC	CAGGTCTACTGTCAGTTCGTCACTGC	IRGSP (2005)
RH7	CTTGCGTTCCGTAGGAGAAG	TGAGTGTAACCCGAAGTGGC	Liu et al. (2015)
W1	TCCTAATCAGCCAATAAATCA	GCAATCTAGTGCACGAACATA	Liu et al. (2015)
C5-indel3678	AAATGTTGCAGAGGAAGATTGG	CACTCCAAATCCTGTCAGTGAA	Yonemaru et al. (2015)
C5-indel3680	GTTGATTAATGCCTGGTTTGGT	TTGTTCCGAGAGGATTACAACC	Yonemaru et al. (2015)
MS10	CAATACGAGAAGCCCCTCAC	CTGAAGGAACACGCGGTAGT	Yang et al. (2004)
RM16535	ACGCGGTAGTCCTCTTCAATGTCG	GGCGCCAACCCTTCCTACTACC	IRGSP (2005)
RM16550	ACAGTACCGCTGCTACTGCTTTGC	TAGATAGCAGCCCAGCCCATGC	IRGSP (2005)
RM16554	GCAACCAAAGTTGGTAACGAGAGC	CCGGCGCAATCTATTAGACACC	IRGSP (2005)
RM16605	CATGGTGGGACTCACATATACC	AAGAACTGTGCTGCAGATAACC	IRGSP (2005)
C5-indel3729	GCTCCGCATCTACTTCAAGTTT	CCAAAGGTTTACACGAGTGCAT	Yonemaru et al. (2015)
C5-indel3743	CTCAACAATGGCCACACAAA	CAATGGCCAATCTAAACCCTAA	Yonemaru et al. (2015)
C5-indel3757	CACAAGAAATCAACCATGTCGT	CAGGTGGTTGGAATTAGCTAGAG	Yonemaru et al. (2015)
RM16626	ACATGATTGCTGGCTTGCTTACC	GCCACGCAGTGTTGTTTCAGC	IRGSP (2005)
RM307	GTACTACCGACCTACCGTTCAC	CTGCTATGCATGAACTGCTC	McCouch et al. (2002)
